# Optimization of alkaline extraction of hemicellulose from sweet sorghum bagasse and its direct application for the production of acidic xylooligosaccharides by *Bacillus subtilis* strain MR44

**DOI:** 10.1371/journal.pone.0195616

**Published:** 2018-04-10

**Authors:** Lusha Wei, Tongjing Yan, Yifei Wu, Hui Chen, Baoshan Zhang

**Affiliations:** 1 College of Food Engineering and Nutritional Science, Shaanxi Normal University, Xi’an, Shaanxi, China; 2 Department of Life Science, Northwest University, Xi’an, Shaanxi, China; 3 College of Forestry, Northwest A&F University, Yangling, Shaanxi, China; Universidad Nacional Autónoma de México, MEXICO

## Abstract

As predominant components of hemicelluloses in grasses, methylglucuroarabinoxylans (MeGAX_n_) are sources for the production of acidic xylooligosaccharides (U-XOS). *Bacillus subtilis* MR44, an engineered biocatalyst to secrete only the XynC xylanase and Axh43 arabinoxylan hydrolase is capable of processing MeGAX_n_ to exclusively U-XOS. The present studies are directed at the explosion on direct alkaline extraction serving for production of U-XOS. Response Surface Methodology was used to optimize xylan extraction conditions on the sweet sorghum bagasse to achieve maximum hemicelluloses yield. The optimized condition was as follows: extraction time of 3.91 h, extraction temperature of 86.1°C, and NaOH concentration (w/w) of 12.33%. Crude xylan extracted with NaOH revealed a compositional analysis of xylose (79.0%), arabinose (5.3%), glucose (1.7%), lignin and ash (5.6%). After neutralization this xylan preparation supported growth of MR44, processing MeGAX_n_ from sweet sorghum and accumulating U-XOS. The quality of U-XOS produced by MR44 using alkaline-treated sweet sorghum bagasse was comparable to that obtained from purified MeGAX_n_. Overall, the present study demonstrates that direct alkaline treatment of sweet sorghum bagasse is useful to improve the bioavailability of MeGAX_n_ for MR44-mediated conversion to U-XOS with average degrees of polymerization of 11–12, providing alternative resources with applications in nutrition and human and veterinary medicine.

## Introduction

Glucuronoarabinoxylans (MeGAX_n_) are heteroxylans which have a backbone of β-(1–4)-xylosyl residues variably modified with α-1, 2 methylglucuronate or α-1, 3 linked L-arabinofuranose residues. These are the major polysaccharides comprising the hemicellulose fraction of monocots, including important agriculture crops, e.g. sorghum. Sweet sorghum is a promising energy crop due to its high sugar content and has been recognized as a renewable, widely available resource and relatively inexpensive [1, 2]. Acidic xylooligosaccharides (U-XOS, where U represents uronate) are oligosaccharides produced during the hydrolysis of xylan-rich hemicelluloses from dicots and monocots by glycoside hydrolases of family 30 [3, 4]. As we know the type of substituting residues, their degree and pattern of substitution along the xylan backbone are structural features that determine the physicochemical and biological properties of XOS [5]. The production of oligosaccharides with a desired DP range has been achieved using specific endoxylanases and conditions for the depolymerization process [6], while control of production of oligosaccharides with a desired DP range can be difficult during hydrolyzing xylan by chemical hydrolysis. It is necessary to find out an efficient way for production of U-XOS products with certain degrees of polymerization (DP) as we utilized sweet sorghum bagasse with engineered *B*. *subtilis strain* which exclusively produce U-XOS with a MeG substitution occurs on a xylose residue penultimate to the reducing terminal xylose [7].

As the compositions of plant cell wall complexity, biomass conversion is always expensive with multiple steps. In order to achieve effective bioprocessing of hemicelluloses from lignocellulosic resources for utilization by *B*. *subtilis* strains, a series of preliminary studies have been conducted to determine optimal conditions for extraction and solubilization of MeGAX_n_ from lignocellulosic biomass. Treatment of milled material with a 12% NaOH solution for 4 h at 80°C extracted 88–90% of the pentosans from sweet sorghum bagasse and wheat straw [8]. Other reports showed the extraction with solutions of alkali in ethanol were effective for carbohydrates from plant cell walls. For instance, the extraction of *Caragana sinica* with 1% sodium hydroxide containing 70% ethanol achieved 35% original hemicellulosic polymers [9]. It should be aware that alkaline solutions of sodium, potassium and lithium hydroxide are suitable for extraction of hemicelluloses from lignocellulosic biomass, but there was other report showed the preferred alkaline solution was potassium hydroxide as potassium acetate formed during the neutralization process of the alkaline extracted is more soluble in ethanol used for precipitation than other acetates [10]. Therefore, it is necessary to evaluate those methods of extracting hemicelluloses from sweet sorghum bagasse for growth of *B*. *subtilis* stains and production of U-XOS.

The complete utilization of glucuronoarabinoxylans requires a combination of glycoside hydrolases, including endoxylanases, α-glucuronidases, xylosidases and arabinofuranosidases, to release fermentable sugars. *B*. *subtilis* 168 and related species are xylanolytic and utilize MeGX_n_ and MeGAX_n_ for growth and fermentation. *B*. *subtilis* strains contain genes encoding XynA and XynC endoxylanases, and these are the only activities secreted that process these polysaccharides to xylooligosaccharides. XynC xylanse is exclusively active on glucuronoxylan [11]. The combined action of these enzymes on MeGX_n_ generates β-1, 4-xylobiose and β-1,4-xylotriose that are assimilated by ABC (ATP-binding cassette) transporters and metabolized, along with the aldouronate. MeGX_3_ in which MeG is α-1, 2-linked to the middle xylose in β-1,4-xylotriose. This MeGX_3_ is not metabolized and accumulates in the medium, representing as much as 40% of the xylose in the hemicellulose fraction of dicots. Strain MR44 with a deletion in the gene encoding the XynA xylanases secretes only the XynC xylanase which converts MeGX_n_ from sweetgum wood exclusively to acidic xylooligosaccharides ranging in degree of polymerization from 5 to 18 with an average of 6.8 [12]. Meanwhile, strain MR44 was engineered to secrete a XynC xylanase and a Axh43 arabinoxylan hydrolase as the only xylanolytic enzymes accumulated U-XOS with an average DP of 11–12 and a single 4-O-methyl-glucuronic acid α-1,2-linked on average to one of 11–12 xylose residues each penultimate to the reducing terminal xylose in purified sweet sorghum xylan. Axh43 removed arabinose side chain linked to the xylan to generate methylglycuronoxylans (MeGX_n_) that were following processed by XynC to U-XOS [7].

The objective of this research work was to explore on direct substrate extraction method serving for MR44 growth and production of U-XOS. For this purpose, the Response Surface Methodology (RSM) technique was applied to optimize the process for extraction of crude xylan from sweet sorghum bagasse. Subsequently, we evaluated the bioconversion of extracted crude xylan substrate to U-XOS by MR44 strain for the production of U-XOS.

## Materials and methods

### Materials

Sweet sorghum (*Sorghum bicolor* (L.) Moench) bagasse was obtained from Tianjiao farm in Xi’an, China. The bagasse was air-dried and ground to 1 mm mesh size.

Xylose, xylobiose, xylotriose and xylotetraose were from Sigma-Aldrich Corporation (St. Louis, MO). All other chemicals and reagents were obtained from Sigma Company (USA), Merck (Germany) and Thermo Fisher Scientific (UK).

### Extraction of xylan from sweet sorghum bagasse

Three different strategies were applied in order to evaluate maximum amount of xylan from sweet sorghum bagasse. In the first method, as reported by Don et al [13], 5 g of sweet sorghum bagasse was soaked in 12% NaOH (w/w) and incubated at 80°C in an oven for 4 h. The supernatant fraction was centrifuged at 8500 x g for 20 min, neutralized to pH 7.0 with concentrated HCl and dried at 60°C in the oven to constant weight. The pellets were weighed and powdered in a mixer and stored at room temperature for U-XOS production by *B*. *subtilis* strains. In the second method, sweet sorghum bagasse was extracted with 2% KOH (w/w) and incubated at 75°C in an oven for 3 h. The processes that follow were the same as the previous description. The third method was similar to the second one but with slight modification. Sweet sorghum bagasse was blended with 2% KOH (w/w) containing 60% ethanol and incubated at 75°C in an oven for 3 h, the following process was the same as the previous description. The second and third methods were adapted from Sun et al. [14] with slight modification.

### Surface response design on NaOH extraction of xylan and composition analysis

The effect of several extraction parameters on crude xylan from sweet sorghum was carried out in batch systems. The effects of temperature, NaOH concentration and extraction time were optimized with surface response design methodology in the DESIGN EXPERT 8.0.6 software. Three different extraction temperatures (70, 80 and 90°C), three NaOH concentrations (w/w) (10%, 12%, and 14%), and three different extraction times (hours) (3, 4 and 5 h) were tested. The response of the experimental design was based on the reducing sugar released. The Box-Behnken design (BBD) was employed for designing the experimental data.

The crude xylan obtained from sweet sorghum bagasse was autoclaved with 4% sulfuric acid at 120°C for 1 h to deduce the monomer composition [15, 16], then the hydrolysate was filtrated and neutralized using 1M NaOH. Fifty-fold dilution of the sample was injected into the HPLC with an Agilent 1200 system. 5 μL of samples were injected into a BioRad Aminex HPX 87P column (300 × 78 mm) (Hercules, CA, USA) equilibrated and eluted with 5 mM H_2_SO_4_ at 0.6 ml/min at 60°C and detected by a refractive index detector at 210_nm_ (Agilent, USA) [17]. The concentrations of respective sugars (xylose, arabinose, mannose and glucose) were quantified after comparing to integrated peak areas of standards. The residue was used to measure acid insoluble lignin. Ash content was washed with DI water several times and dried in the vacuum oven at 60°C until constant weight.

### *B*. *subtilis* strains and growth media

*B*. *subtilis* strains 168 and MR44 were obtained from the Department of Microbiology and Cell Science, University of Florida. The MR44 (168, *ΔxynA*-Spc) strain has been described in ref [12]. *B*. *subtilis* strains were cultured at 37°C in LB broth (Lennox L broth) supplemented with low-salt formula (RPI Corp.). Spizizen’s medium [18] was used for cultivation on sorghum carbohydrate substrates. From an 18 h standing LB culture (1.0 ml of LB with antibiotics: MR44 (spectinomycin, 100 μg/ml), 0.03 ml were inoculated in 1.0 ml of the same medium and incubated for 3 h at 37 °C with shaking. Cultures grown to late log phase (O.D.600_nm_ 0.6–0.7) were inoculated into 20 ml Spizizen’s minimal media with 0.5% alkali extracted substrate to give an O.D.600_nm_ of around 0.03. Cells were cultured at 37°C with gyratory shaking (200 rpm).

### Preparation and analyses of accumulated XOS and U-XOS

Cultures with 0.5% crude sweet sorghum xylan as the carbon source in modified Spizizen’s medium containing 0.1% yeast extract were incubated at 37°C with gyratory shaking (200 rpm) for 48 h. 10 μL of samples from the cultures were spotted onto TLC plates directly for identification of the accumulated XOS and U-XOS. Cells were removed by centrifugation (10,000 x g, 8 min, 4°C), and the supernatants was clarified by filtration through a cellulose acetate membrane filter (10,000 MCO). The filtrate (10K) was dialyzed twice against 4 L of water with dialysis tubing (1000 MCO), lyophilized and analyzed by ^1^H NMR spectroscopy as described below.

### ^1^H NMR analysis of crude xylan hydrolysis products

Sample preparation for ^1^H NMR preparation were as previously described [3]. Samples dissolved in D_2_O were transferred to 5 mm od. NMR tubes (Wilmad, Buena, NJ) (internal acetone ^1^H (CH_3_) at 2.225 ppm). ^1^H-NMR spectrum was recorded on a Bruker AV III NMR spectrometer operating at 400.13 MHz using 5 mg of hydrolysis products in 1.0 mL of D_2_O at the Department of Chemistry, Shaanxi Normal University. NMR data were analyzed using the MestReNova software. The chemical shifts for ^1^H atoms were assigned on the basis of previous literature [19].

## Results

### Composition of raw sweet sorghum bagasse

The composition of the sweet sorghum bagasse is depicted in Table 1. It can be seen that the dehydrated sweet sorghum bagasse contained (wt/wt, dry basis) 42.8% cellulose, 26.3% hemicelluloses, and 20.2% lignin. These results are consistent with those previously published for sorghum bagasse [20].

**Table 1:** Compositional analysis of raw bagasse samples.

Component	Amount(%) ± SD^a^
Glucan	42.8 ± 1.6
Xylan	26.3 ± 0.9
Arabinan	2.2 ± 0.6
Acetyl groups	0.08 ± 0.004
Lingin (acid insol + acid sol)	20.2 ± 0.8
Ash	2.2 ± 0.4

^a^ composition is expressed on dry basis.

### The effects of different solvents on alkali extractions

As previously reported the application of alkali, such as NaOH and KOH, is the preferred method for the extraction and solubilization of hemicelluloses fractions because of their ability to disrupt hydrogen bonds and hydrolyze ester bonds that results in the swelling of cellulose and decreasing crystalinity of cellulose [8]. Moreover, other researchers found that extraction with solutions of alkali in ethanol is effective for disrupting the recalcitrant nature of the plant cell wall [9]. In the present study, xylan from the sweet sorghum bagasse was extracted through application of NaOH and KOH (with or without ethanol). It can be seen in Table 2 that treatment of NaOH and KOH was effective for fractionating the xylan from the lignocellulosic complex of sweet sorghum bagasse, yielding 33.3 ± 0.7% and 21.4 ± 0.8%, respectively, of initial biomass on a dry weight basis. Treatment of KOH with 60% ethanol is, however, less effective.

**Table 2:** The effects of alkali and extraction conditions on recovery of crude xylan from sweet sorghum bagasse.

Extraction conditions	Recovery of crude xylan (g/100 g of bagasse)
12% NaOH, 80°C, 4 h	33.3 ± 1.1
2.0% KOH, 75°C, 3 h	21.4 ± 0.8
2.0% KOH, 60% ethanol, 75°C, 3 h	7.6 ± 0.9

**Fig 1:**
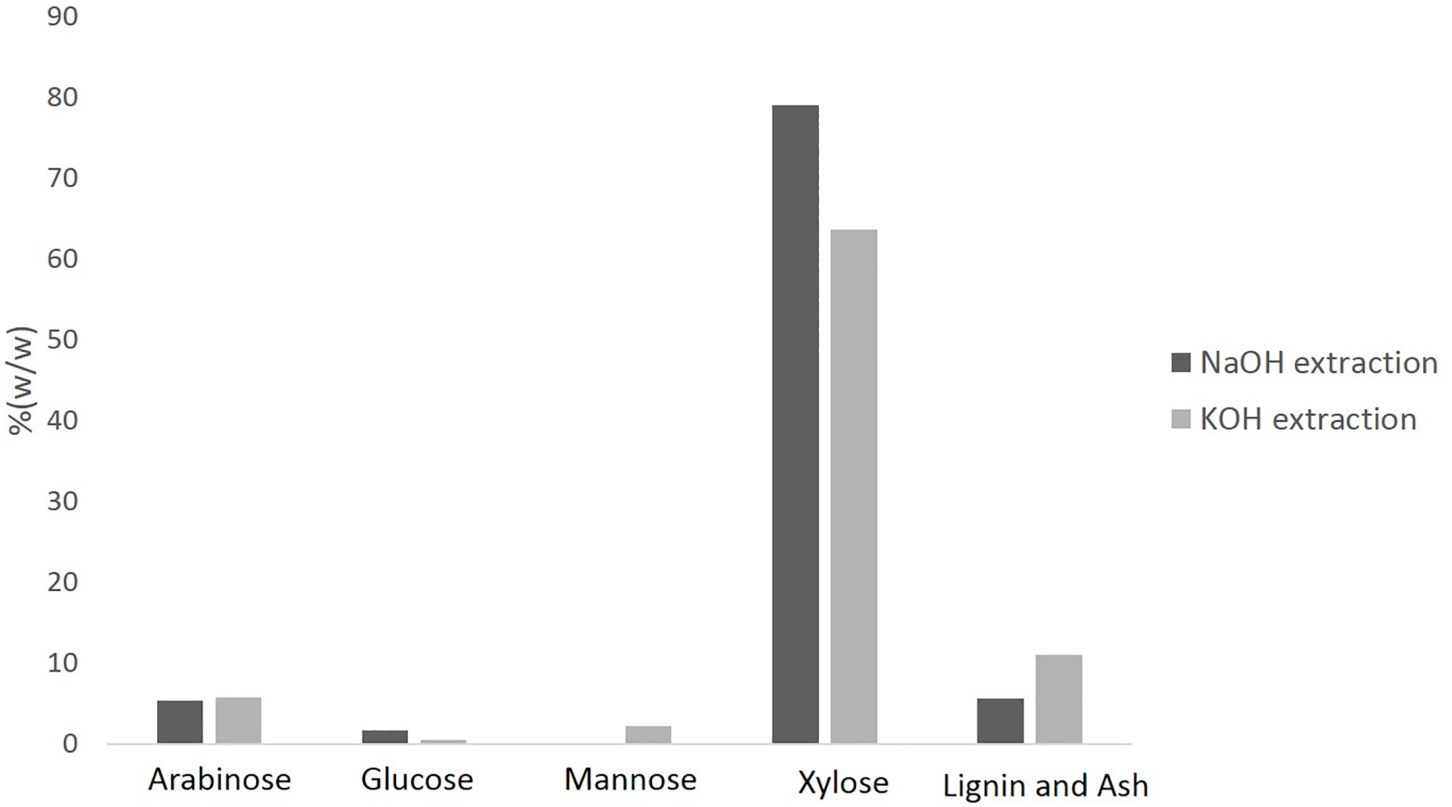
The contents of neutral sugar and others composition (relative %, w/w) of the alkali extraction obtained from sweet sorghum bagasse with treatment of NaOH and KOH.

**Fig 2:**
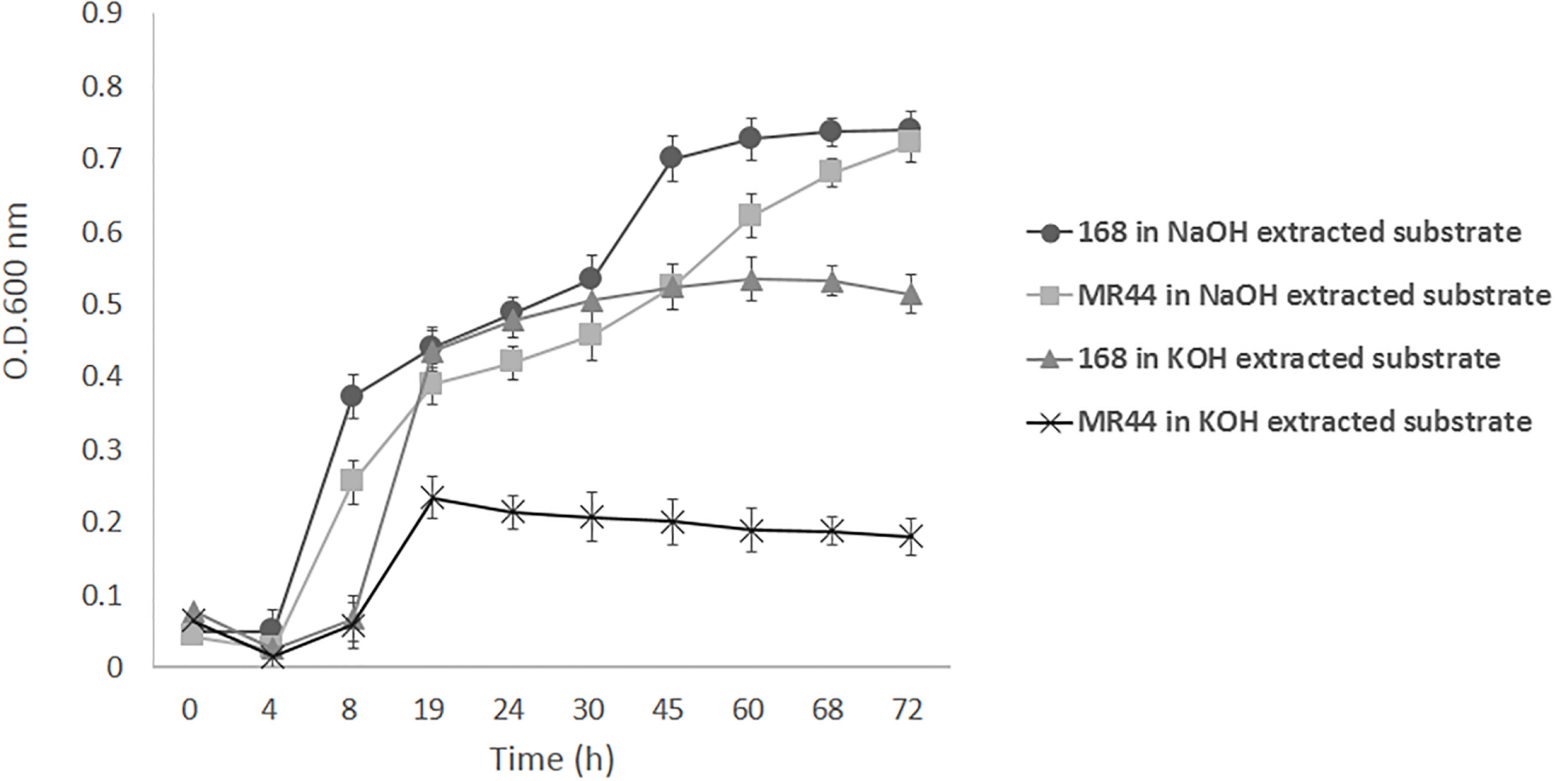
Growth comparison of *B*. *subtilis* 168 and MR44 in substrates with different treatments.

The neutral monosaccharide composition and the content of lignin and ash after NaOH extraction and KOH extraction were given in Fig 1. NaOH extraction contained a significant amount of xylose (79.0%) together with small amounts of arabinose (5.3%), glucose (1.7%), non-detectable mannose, and lignin and ash (5.6%). In comparison, KOH extraction contained a significant amount of xylose (63.6%) together with small amounts of arabinose (5.7%), mannose (2.2%), glucose (0.5%), and lignin and ash (11.0%). The results indicted xylose was the predominant sugar component of either NaOH extraction or KOH extraction, but NaOH may be more efficient on releasing xylan from lignocellulosics with less lignin dissolution. Moreover NaOH extraction contained more xylose than KOH extraction may also explan why *B*. *subtilis* 168 and MR44 grew more rapidly in NaOH extraction than in KOH extraction (Fig 2). Lignin level also appears as a determinant factor to cell growth. Taken together, the NaOH extraction produces higher yield of xylose which is more suitable for cell growth and U-XOS production.

### Cell growth in direct alkaline extractions

To determine which substrate is more suitable for cell growth, *B*. *subtilis* 168 and MR44 were separately inoculated into NaOH and KOH pre-treated extracts. *B*. *subtilis* 168 and MR44 cultures were grown with xylan prepared by either the 12% NaOH 80°C or the 2.0% KOH 75°C extracts as described in Table 2. In Fig 2, the 168 parent strain, which can secrete both XynA and XynC, grew to a higher turbidity than MR44 which is engineered secrete only the XynC on the KOH extract and the NaOH extract. Growth difference is expected since the XynA is required for the generation of X_2_ and X_3_, the predominant products derived from the MeGAX_n_ when assimilated and metabolized. The arabinose released by the arabinoxylan aribinofuranosidase Axh43 is expected to be assimilated and metabolized by the MR44 as well as the 168 strain. Both strains shows higher growth on the NaOH extract than the KOH extract, suggesting the NaOH extract is releasing more carbohydrate for metabolism. The NaOH treatment may be rendering some of the cellulose accessible to the action of glucanase that is secreted by *B*. *subtilis* 168 [21, 22].

### Model fitting and statistical analysis

As *B*. *subtilis* strains showed greater growth in NaOH extraction, we then optimized the operating parameters of NaOH extraction. To examine the combined effects of the variables extraction time (min), Temperature (°C)and NaOH concentration (%) on the crude xylan extraction, a BBD of 17 runs leading to a set of experiments was performed randomly, with five center points (Table 2 treatment 13–17) at the centre of the design were used to allow for estimation of pure error. Table 3 listed the whole design consisted of 17 experimental points. The triplicates were performed at all design points in randomized order. This design is being represented by a quadratic polynomial regression model.

**Table 3:** Box-Behnken experimental design with the independent variables in coded units and response values for the extraction rate of the polysaccharides.

Run	Coded variable levels			Total sugars (mg/g)	
	Time (min) A	Temperature(°C) B	NaOH conc. (%) C	Actual	Predicted
1	0	1	1	28.7	28.85
2	1	0	-1	22.9	23.21
3	1	-1	0	30.6	30.56
4	-1	0	-1	22.5	22.36
5	0	0	0	33.3	33.36
6	-1	-1	0	27.6	27.76
7	-1	0	1	27.5	27.44
8	0	1	-1	24.6	24.57
9	0	0	0	33.1	33.36
10	0	-1	1	26.2	26.23
11	1	0	1	28.4	28.29
12	0	0	0	33.6	30.56
13	1	1	0	32.2	32.04
14	0	0	0	33.5	33.36
15	-1	1	0	33.1	33.36
16	0	-1	-1	20.5	20.35
17	0	0	0	33.3	33.36

By applying multiple regression analysis the experimental data, the predicted response Y (the yield of total sugar) was obtained using the following equation:

$$\text{Y}=33.36+0.43\text{A}+1.71\text{B}+2.5\text{C}-0.97\text{AB}+0.12\text{AC}-0.40\text{BC}-1.08{\text{A}}^{2}-1.41{\text{B}}^{2}-6.95{\;\text{C}}^{2}$$ (1)

where A, B, and C are the coded variables for extraction time, extraction temperature and NaOH concentration, respectively.

This equation was tested for adequacy by the analysis of variance (ANOVA). In a quadratic polynomial model, the *F*-ratio in general is the ratio of the mean square error to the pure error obtained from the replicates at the design center. The significance of the *F*-value is shown in the *p*-value column (95% confidence level) and the degrees of freedom (DF) in the model. Thus, the effects lower than 0.05 in this column are being considered significant [23]. Table 4 listed the ANOVA for the fitted second-order polynomial model of extraction yields of sweet sorghum xylan. The Model *F*-value of 738.09 and a very low *p*-value (*p* < 0.0001) implies the model is significant. There is only a 0.01% chance that a Model *F*-Value this large could occur due to noise. The lack of fit measures the failure of the model to represent the data in the experimental domain at points which are not included in the regression. The lack of fit *F*-value and *p*-value were1.51 and 0.3401, respectively, which implied it was not significant relative to the pure error and indicated that the model equation was adequate for predicting the yield of sweet sorghum xylan under any combination of values of the variables. The value of R^2^_Adj_ for Eq (1) was 0.9976, which was reasonably close to 1 and implied that only less 1.0% of the total variations were not explained by model [24]. Thus, the model was highly significant and indicated a high degree of correlation between the actual and predicted data.

**Table 4:** Analysis of variance for the fitted quadratic polynomial equation of extraction of total sugars.

Source	Sum of Squares	DF	Mean Square	*F*-Value	Prob > F
Model	307.94	9	34.22	738.09	< 0.0001
Residual	0.32	7	0.046		
Lack of Fit	0.17	3	0.058	1.51	0.3401
Pure Error	0.15	4	0.038		
Cor Tota	308.26	16			
	R^2^ = 0.9989	R^2^_Adj_ = 0.9976			

The significance of each coefficient was determined using *p*-value showing in Table 4. The *p*-value less than 0.0500 indicate model terms are significant, while this values greater than 0.1000 indicate the model terms are not significant. The data in the Table 3 indicated A, B, C, AB, BC, A^2^, B^2^ and C^2^ are significant model terms that affected the yield of sweet sorghum xylan, and there was significant interaction between extraction temperature (B) and NaOH concentration (C). Meanwhile, NaOH concentration (C) was the major factor affecting the yield of sweet sorghum xylan according to *F*-value in Table 5. The reason was attributed to the decrease in the degree of polymerization and crystallinity, increase in internal surface areas, separation of structural linkages between lignin and carbohydrates and disruption of lignin thereby facilitating easy recovery of xylan from lignocellulosic bagasse [25].

**Table 5:** Estimated regression model of relationship between response variables (yield of sweet sorghum xylan) and independent variables (A, B, C).

Variables	Sum of Squares	DF	Mean Square	*F*-Value	*p*-Value
**A**	1.45	1	1.45	31.17	0.0008
**B**	23.46	1	23.46	506.1	<0.0001
**C**	51.51	1	51.51	1111.18	<0.0001
**AB**	3.8	1	3.8	82.03	<0.0001
**AC**	0.063	1	0.063	1.35	0.2836
**BC**	0.64	1	0.64	13.81	0.0075
**A**^**2**^	4.91	1	4.91	105.94	<0.0001
**B**^**2**^	8.31	1	8.31	179.3	<0.0001
**C**^**2**^	203.7	1	203.67	4393.53	<0.0001

### Analysis of response surface

**Fig 3:**
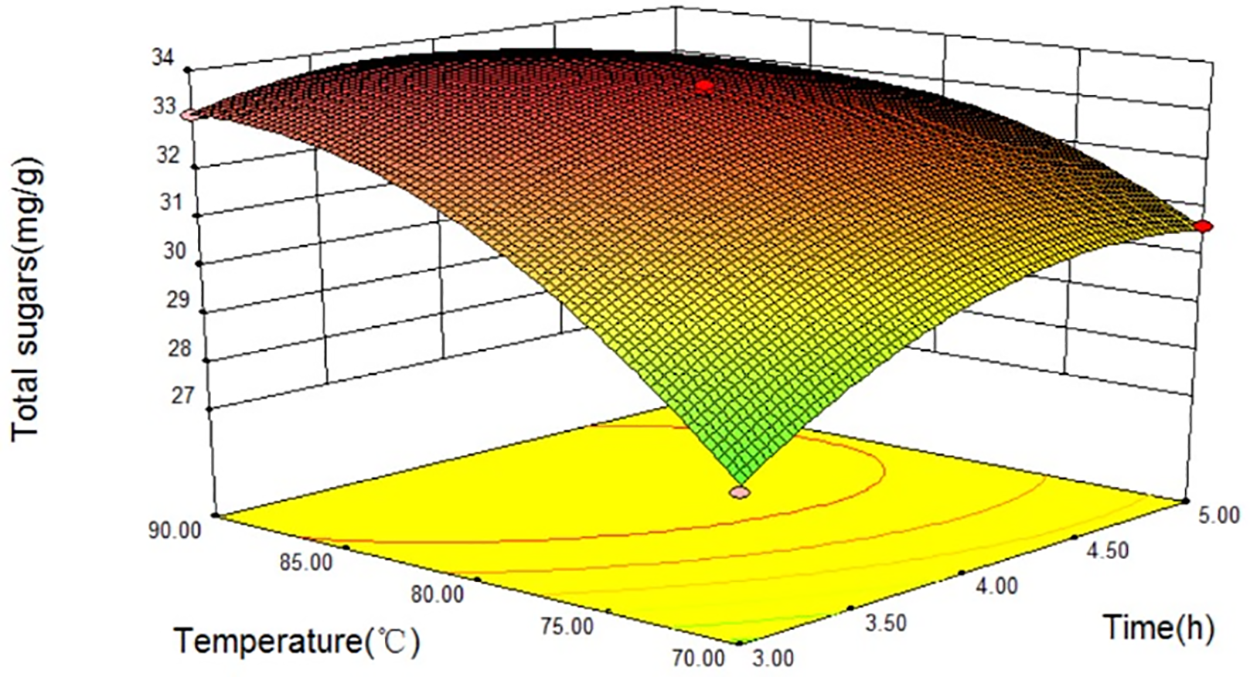
Response surfaces for total sugars released from sweet sorghum bagasse extracted with NaOH at different extraction temperatures and extraction times.

**Fig 4:**
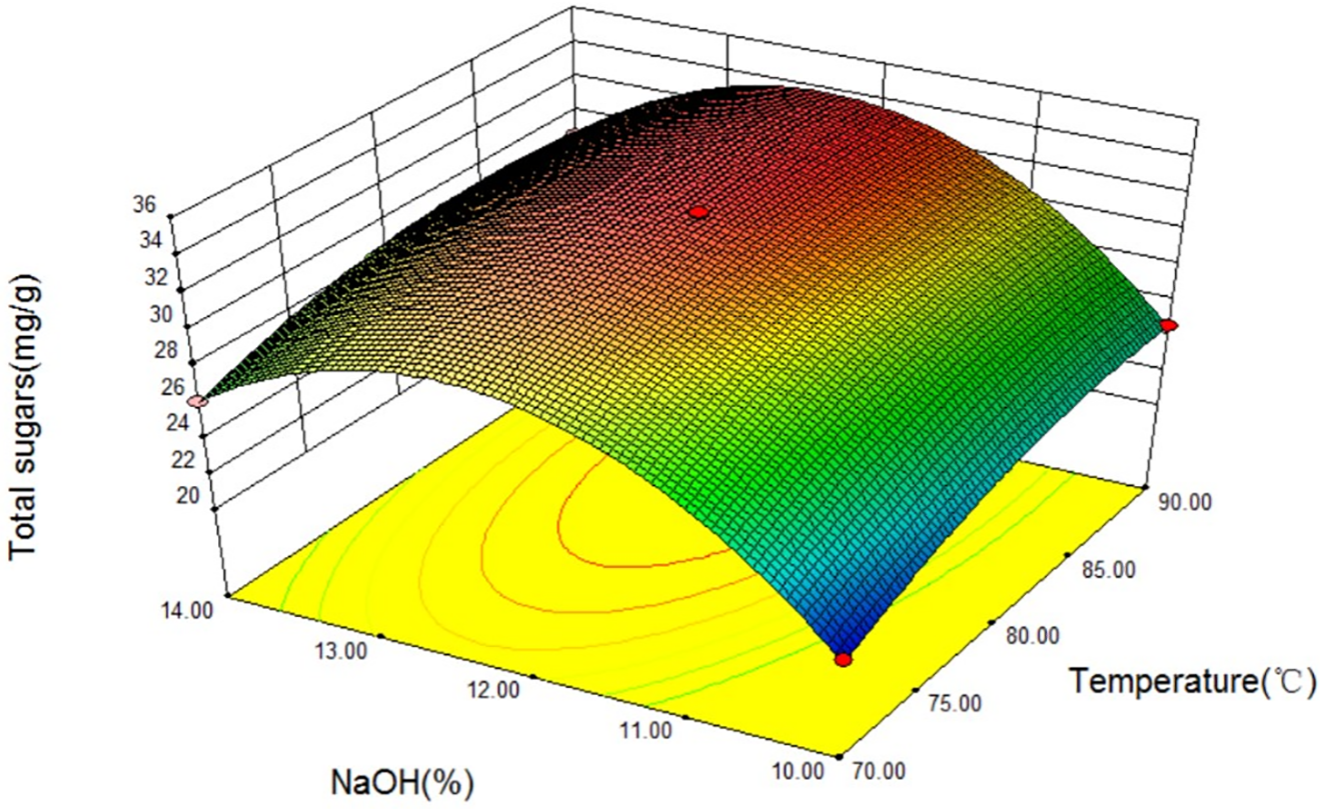
Response surfaces for total sugars released from sweet sorghum bagasse extracted with different NaOH concentrations at extraction temperatures.

**Fig 5:**
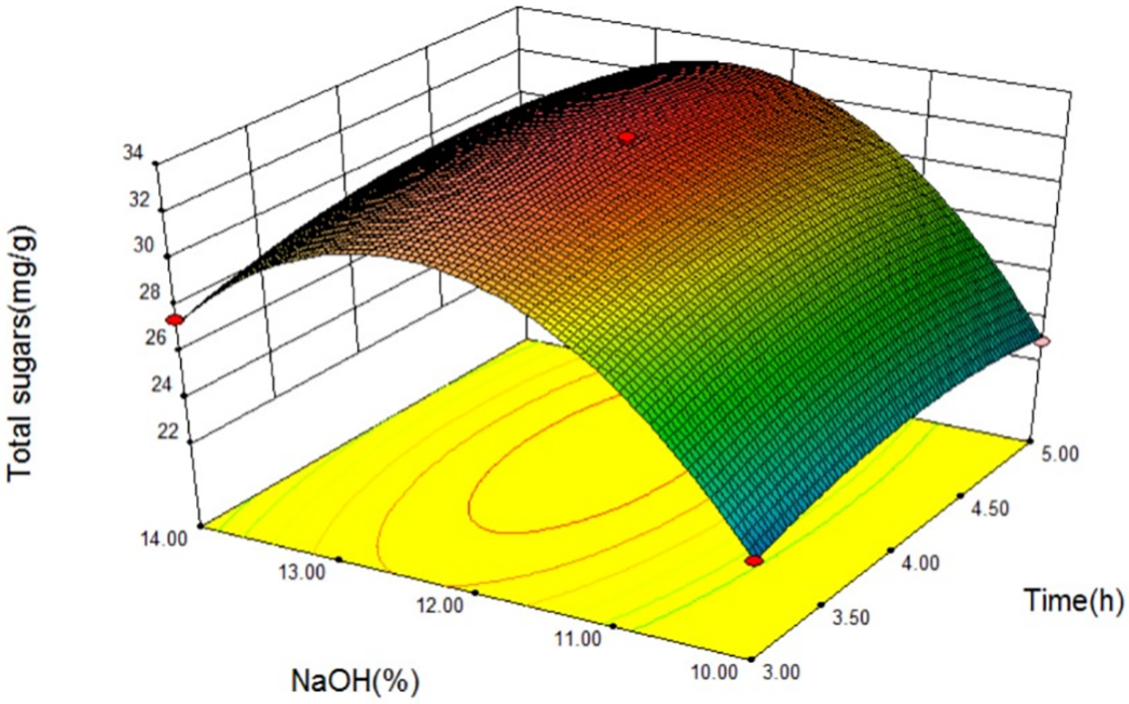
Response surfaces for total sugars released from sweet sorghum bagasse extracted with different NaOH concentrations at different extraction times.

The relationship between independent and dependent variables was illustrated in a 3D response surfaces generated by the model for productivity of sweet sorghum xylan (Figs 3–5). It is possible to obtain response surfaces and the mathematical correlations that describes the extraction behavior of the sweet sorghum bagasse. It is clear that the yield of sweet sorghum xylan was sensitive to minor alterations of these test variables (extraction time, extraction temperatureand NaOH concentration).

The interaction relationships of extraction temperature (B) with extraction time (A) and NaOH concentration (C) on the yield of sweet sorghum xylan were depicted in Figs 3 and 4, respectively, indicating that all three parameters had significant effect on the productivity of sweet sorghum xylan. As we can see in Figs 3 and 4, the extraction time (A) and the extraction temperature (B) had positive effect on the total sugars, while the yield of total sugars slightly changed when NaOH concentration (C) was in the range of 10% to 14%. The yield of polysaccharides was rapidly enhanced with the increasing of extraction temperature (B), reaching at its peak value at 86.1°C, and gradually decreases in higher temperatures. Longer extraction time (A) had positive influence on the total sugars, and had a critical value at 3.93 h when at a constant extraction temperature (86.1 °C).

Fig 5 shows the response surface at various extraction times (A) and NaOH concentration (C). It is demonstrated that sugar yield is directly correlated with extraction time. The response curves were comparatively smooth at lower extraction time, indicating a low effect on yield when NaOH concentration (C) was varied in the range of 10% to 14%. However, the total sugar yield decreased with the increase of NaOH concentration at longer extraction time. This result suggestes that extraction time (A) had a different extent of influence on extracted total sugar yield in different NaOH concentration (C). Higher yields of total sugar were not observed at longer extraction time and higher NaOH concentration at the experimental range. Figs 4 and 5 were shown that the interactions between the NaOH concentration and other two extraction variables did not impact the yield of polysaccharides significantly, even though the NaOH concentration was the major factor affecting the yield of sweet sorghum xylan.

The yield of total sugar was estimated to be 34.08 mg/g at the optimum point [extraction temperature: 86.1°C, extraction time: 3.93 h, and NaOH concentration (w/w): 12.33%]. To reconfirm the adequacy of the model Eq (1), three replicates were tested at the estimated optimum conditions. Total sugar yield attained 33.84 ± 0.35 mg/g supporting that this model Eq (1) is satisfactory and accurate.

### Utilization of NaOH-extracted MeGAX_n_ by *B*. *subtilis* strains

*B*. *subtilis* strains have multiple endoxylanases and a-L-arabinofuranosidase making them capable of converting MeGAX_n_ to U-XOS.

**Fig 6:**
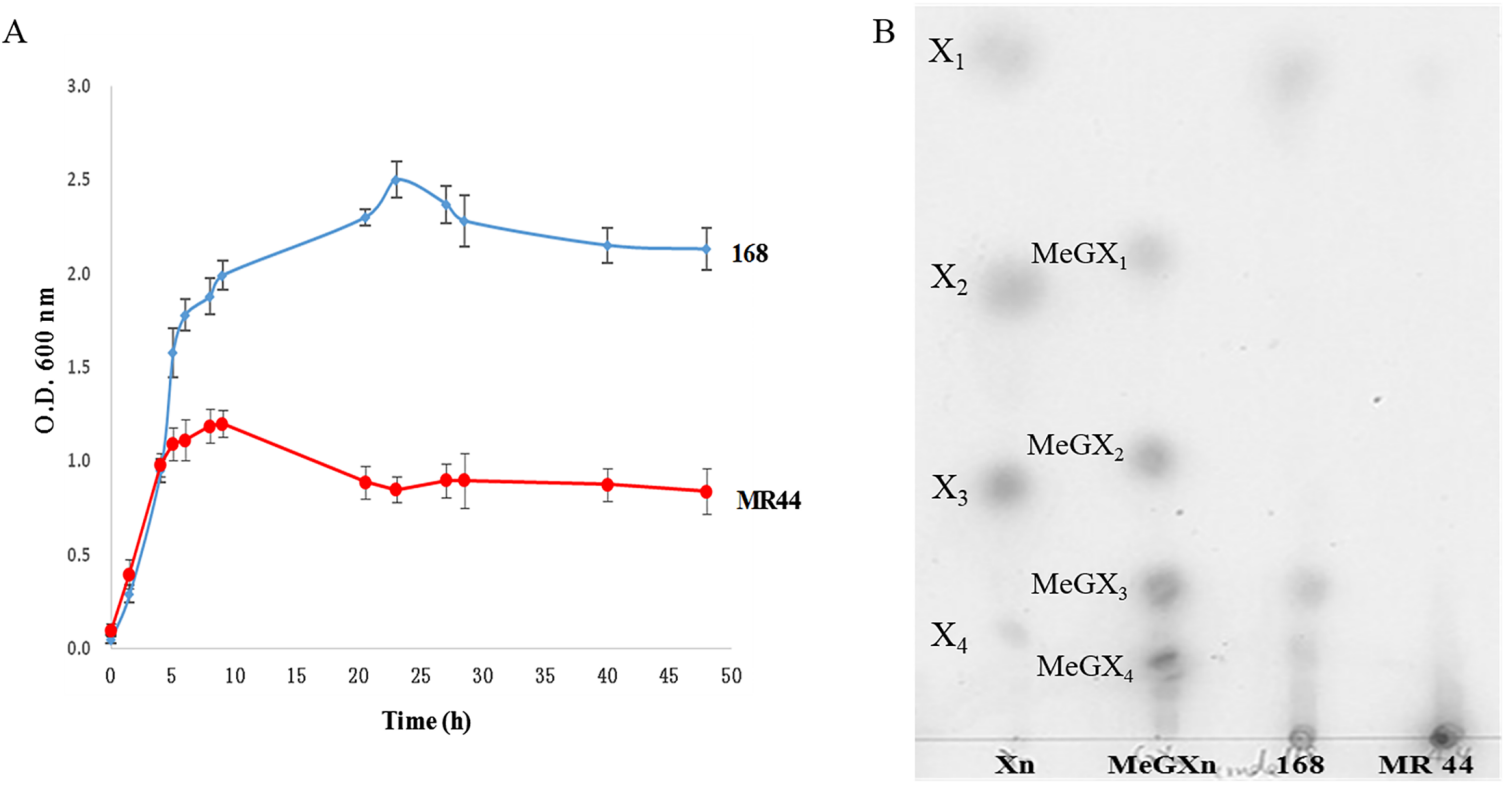
Production of acidic xylooligosaccharides by *B*. *subtilis* strains. (A) Growth comparisons of *B*. *subtilis* 168 and MR44 on crude MeGAX_n_. The experiment was repeated, and the results are given as the means of the two sets of values with error bars. (B) TLC resolution of digestion products accumulated by *B*. *subtilis* 168 and MR44 after 48 h.

Despite the small amounts of lignin present in substrate, *B*. *subtilis* strains 168 and MR44 had still reached stationary phase presumably due to their ability to consumed all polysaccharides they were able to digest (Fig 6). As can be seen in Fig 6A, there was no significant difference of cell growth on pre-treated substrate and on purified xylan in previous study [7], suggesting that small amount of lignin in the substrate does not significantly alter cell growth. *B*. *subtilis* strains 168 secreting (XynA, XynC and Axh43) can process MeGAX_n_ to produced X_2_, X_3_ and arabinose, which are expected to be assimilated and metabolized to other chemicals. MR44 secretes only Axh43 which mediates the release of arabinose that can be further converted by XynC to generate higher DP U-XOS. U-XOS are not assimilated and metabolized by cells causing the accumulation of U-XOS in the medium during the exponential phase of growth.

The utilization of carbohydrate (Table 6) was the greatest for *B*. *subtilis* strain 168 as expected but still incomplete, with 23% remaining at 48 h. Strain MR44 lacking the XynA xylanases consumed 17% of the total carbohydrate suggested the possibility of production of high yield of U-XOS from cell culture and advantage of choosing sweet sorghum bagasse as substrate for utilization. It should be also noted that arabinose released from xylan side chain by Axh43 secrected by both strains also be assimilated by cells and contribute to cell growth. Other sugars may provide some carbohydrate that does not depend upon xylanolytic depolymerization. On the basis of analysis of the sugar composition in hydrolysates of NaOH extraction, glucans may comprise a small amount of the NaOH-treated sweet sorghum as detectable glucose was present.

**Table 6:** Utilization of carbohydrate during growth.

Time (h)	Total carbohydrate (mM xylose equivalents)	
	168	MR44
0	24.1±1.5	24.1±1.5
8	18.9±1.2	23.4±0.4
24	7.6±0.9	21.7±0.9
48	5.6±0.8	20.0±1.1

The accumulation of oligosaccharides in the culture media from each strain were evaluated by thin-layer chromatography (Fig 6B). The absence of detectable arabinose indicates arabinose attached to backbone was released by Axh43 encoded by *axh43* arabinoxylan arabinofuranosidase secreted by both strain168 and MR44 and then assimilated and metabolized for cell growth. Strain 168 accumulated MeGX_4_, which is an expected product of the recombinant XynA, and also MeGX_3_, which is an expected product of the combination of recombinant XynA and XynC xylanase [12]. The much lower levels of these larger U-XOS in the medium of strain 168 cultures indicated the synergistic role that XynA and XynC play in maximizing the production X_2_ and X_3_ for assimilation and growth. MR44 secreting only XynC accumulated larger DP U-XOS as expected which were observed as unresolved products near the origin postion on the TLC plate (Fig 6B).

### ^1^H-NMR spectra of U-XOS products accumulated in cultures

**Fig 7:**
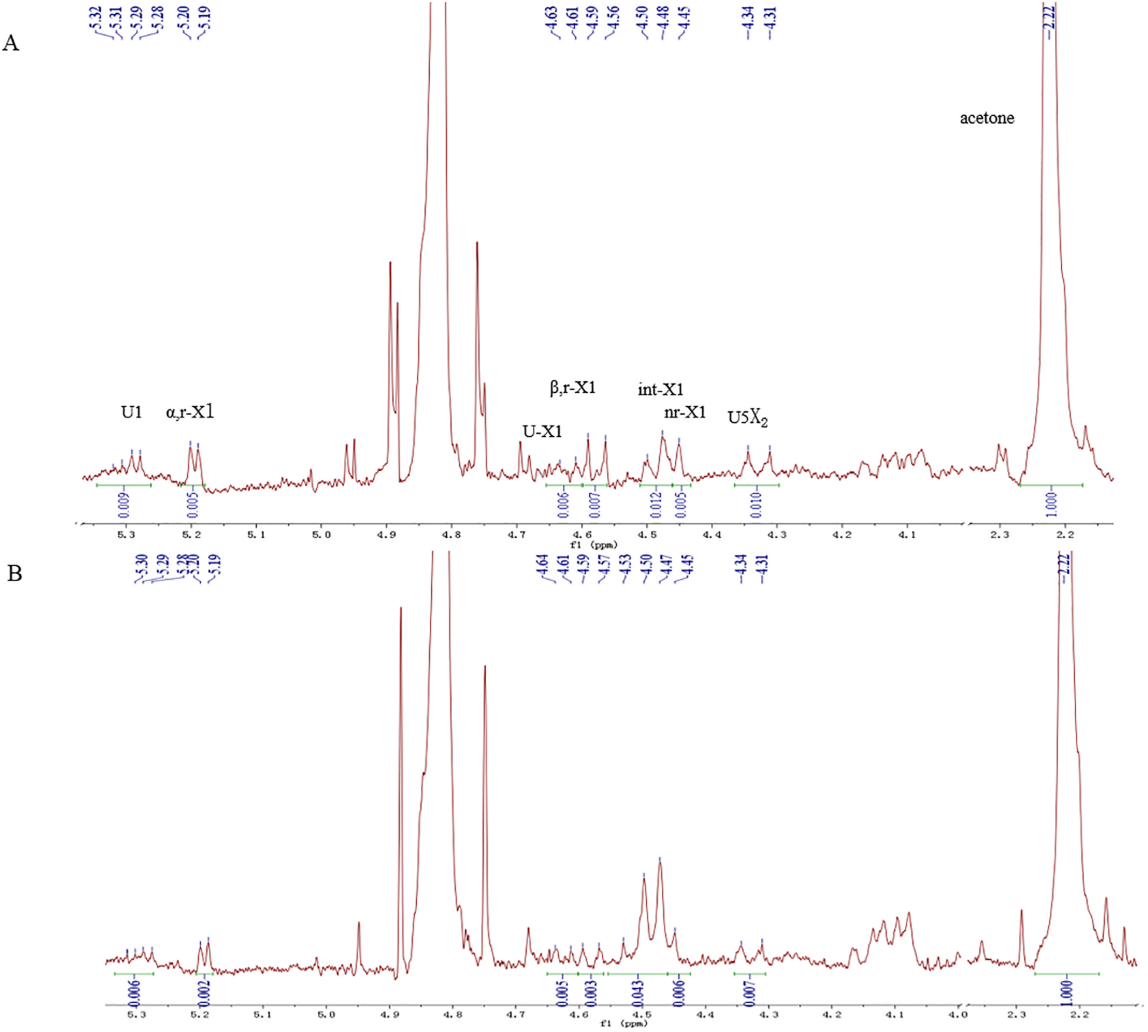
^1^H-NMR analysis of U-XOS products accumulated in cultures of *B*. *subtilis* 168 and MR44 grown on crude xylan extracted directly with NaOH.

^1^H-NMR spectra are allowed to identify the backbone and the side chain of polysaccharides. In this study, *B*. *subtilis* 168 and MR44 were grown to stationary stage and the media were analyzed for accumulated products by ^1^H NMR. The assignment of signals referred to the previous study [13] (Fig 7). As can be seen in Fig 7, signals at 4.2 to 5.3 ppm showed a spectral region of carbohydrate. A broad signals at 5.3 ppm revealed the presence of uronic acid, and the ^1^H linked to uronate C-1 showed a split doublet at 5.28 to 5.32 ppm, showing uronic acid attaching at a xylose penultimate to the reducing terminal xylose. In addition, the relevant anomeric signals of H_1_ for xylose unit appeared in two region: at 5.20 ppm of reducing α-Xyl and at 4.56 ppm β-Xyl units. There was no significant signal at 5.22 to 5.24 ppm indicating the absence of free arabinose in both spectra. Arabinofuranosyl substitutions could have been removed by Axh43 from the xylan backbone and assimilated to support cell growth [26].

In general, the degree of the polysaccharide were in accordance with anomeric signal intensities of individual xylose units. Here we calculated the DP of accumulated U-XOS following the method described in detail in previous study [13]. The ratio of the ^1^H integrals (*α*, r-X1 + U-X1+ *β*, r-X1 + int-X1 + nr-X1)/(*α*, r-X1 + *β*, r-X1) = (0.018 + 0.039 +0.023 + 0.349 + 0.049)/ (0.016 + 0.025) = 11.7, represented the average degree of polymerization, and the ratio of the ^1^H integrals (*α*, r-X1 + U-X1 + *β*, r-X1 + int-X1 + nr-X1)/U1 = (0.018 + 0.039 +0.023 + 0.349 + 0.049)/0.039 = 12.3, represented the average degree of substitution of xylose residues with MeG. The results showed that U-XOS with average degree of polymerization 11–12 with 4-O-methyl-glucuronic acid *α*-1,2-linked on average to one of 11–12 xylose residues which was agreed closely with U-XOS produced on purified xylan in previous study [7].

## Discussion

The present study demonstrates that crude xylan extracted directly from plant tissue using NaOH is potential to provide substrate for the production of acidic oligosaccharides by *B*. *subtilis* strain MR44. The presence of small amount of lignin did not show significant effect on cell growth and production of U-XOS by MR44 strain. Other saccharides that may be present, e.g. arabinose α-1, 3 linked to xylose in the chain, glucose in oligosaccharides released from cellulose, are consumed by MR44 strain, leaving U-XOS. The accumulated U-XOS with an average DP of 11–12 on crude xylan was agreed closely with U-XOS produced on purified xylan in previous study [7]. Purified U-XOS may be achieved by ion exchange or size-exclusion chromatography on the basis of size alone with lignin removed [7].

Meanwhile, we evaluated different alkali extraction methods. The employment of NaOH treatment can gain more xylan from sweet sorghum bagasse compared to the KOH, and *B*. *subtilis* strains showed better growth on NaOH extracted xylan.

Response Surface Methodology (RSM) was applied to estimate and optimize the experimental variables- extraction time (h), extraction temperature (°C), and NaOH concentration (w/w). Model for the amount of total sugars released in sweet sorghum bagasse was gained and could be great employed to optimize hemicellulose extraction from sweet sorghum bagasse by NaOH extraction. The optimal extraction conditions for total sugar from sweet sorghum bagasse were determined as follows: extraction time 3.91 h, extraction temperature 86.1°C, and NaOH concentration (w/w) 12.33%.

## Supporting information

S1 Dataset Growth comparison in different substrates. (XLS)

S2 Dataset Composition of the alkaline extractions. (XLSX)

S1 Fig Surface response design. (PNG)

S2 Fig ^1^H-NMR analysis of 168 culture. (PNG)

S3 Fig ^1^H-NMR analysis of MR44 culture. (PNG)
