# Supplementary figures and images for: Optimization of alkaline extraction of hemicellulose from sweet sorghum bagasse and its direct application for the production of acidic xylooligosaccharides by Bacillus subtilis strain MR44
Source: PLoS One. 2018 Apr 10;13(4):e0195616. doi: 10.1371/journal.pone.0195616 (PMC5892927; doi:10.1371/journal.pone.0195616)

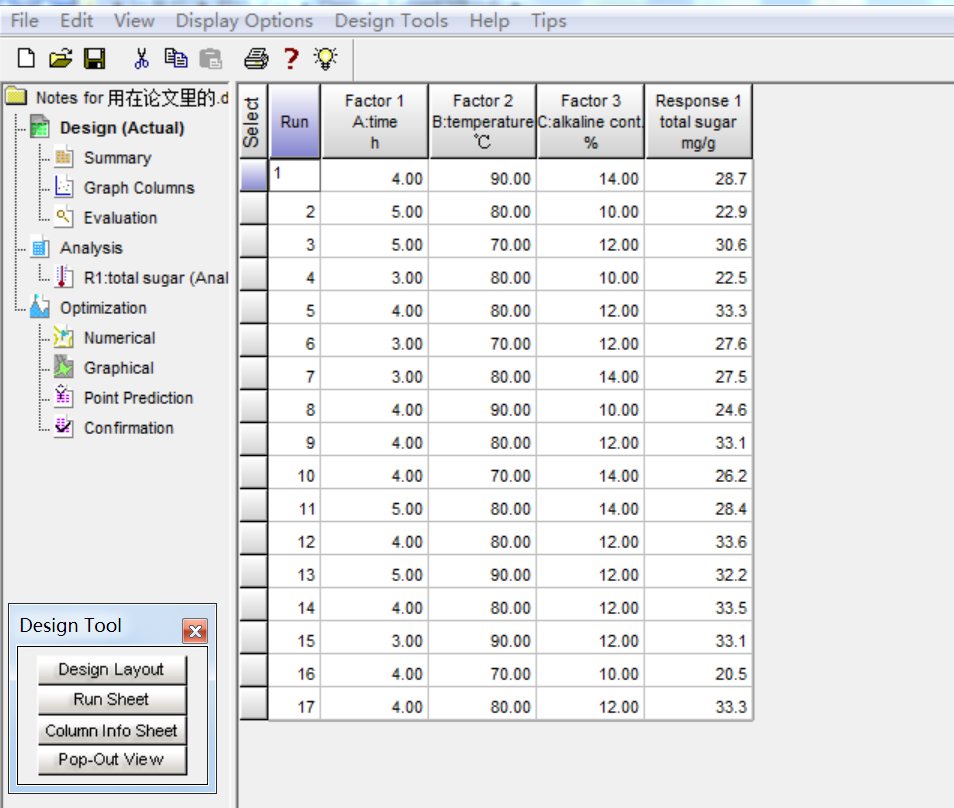

Supplement: S1 Fig — (PNG) [file pone.0195616.s003.png]

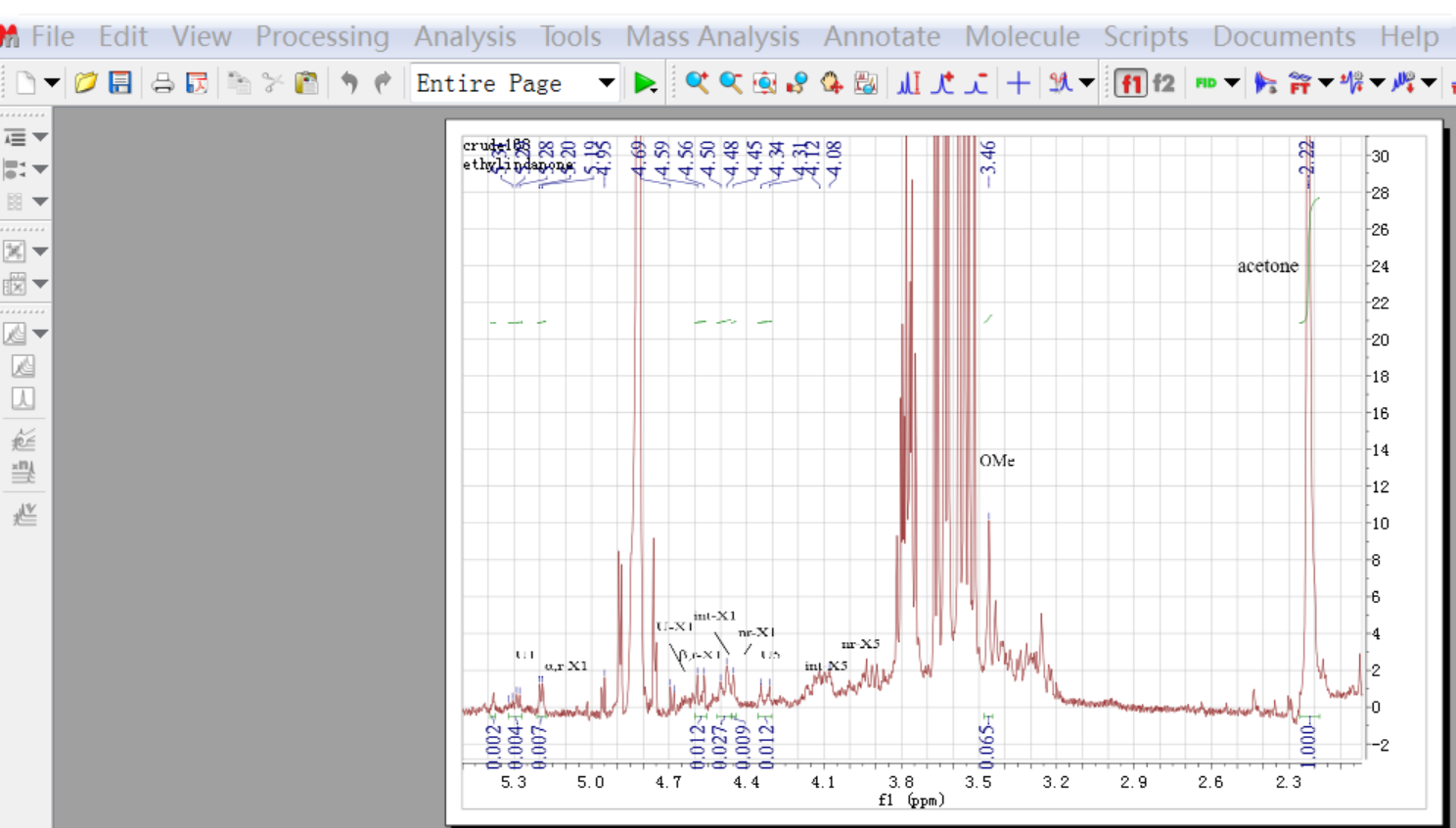

Supplement: S2 Fig — (PNG) [file pone.0195616.s004.png]

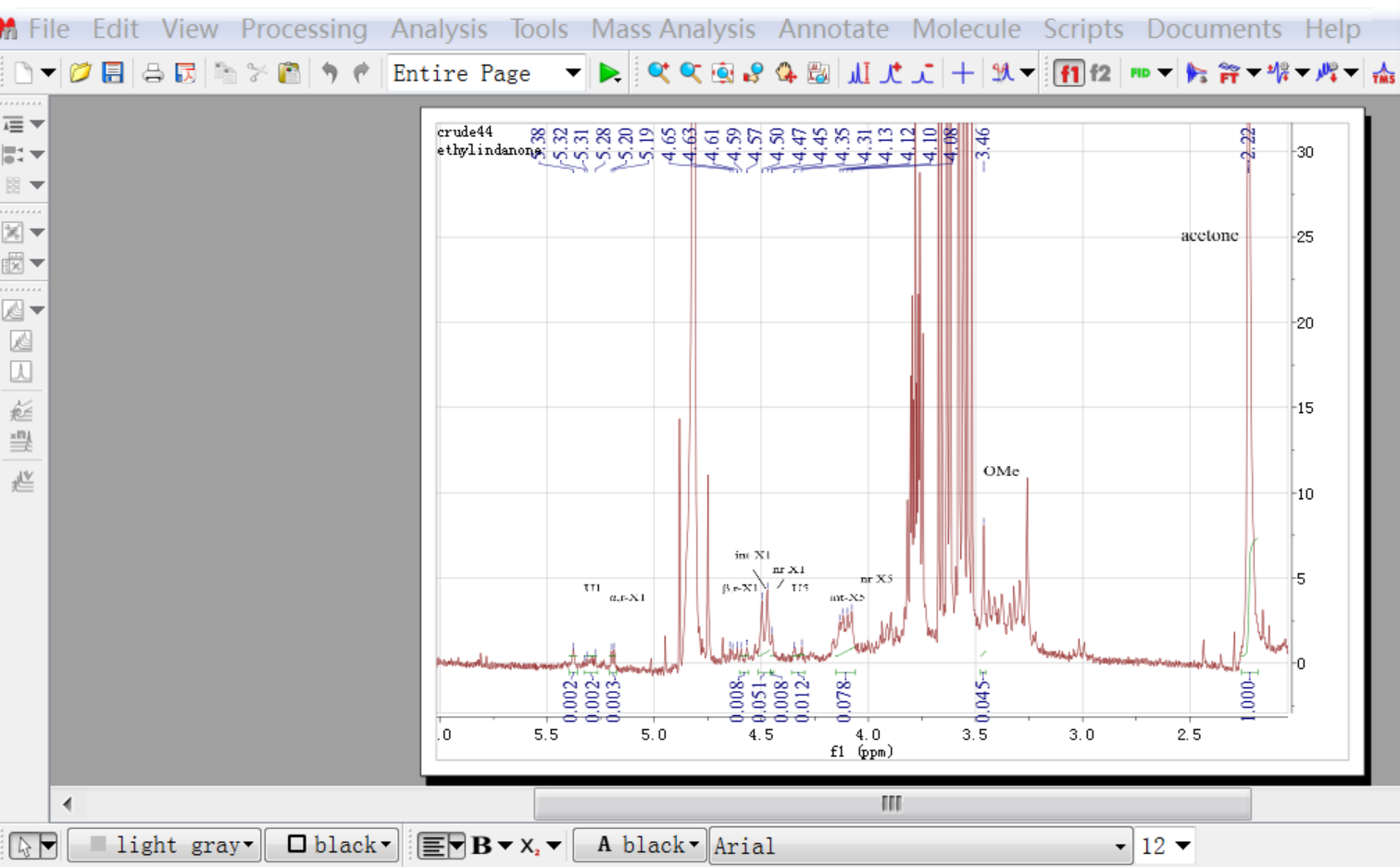

Supplement: S3 Fig — (PNG) [file pone.0195616.s005.png]
